# System-Wide and Group-Specific Health Service Improvements: Cross-Sectional Survey of Outpatient Improvement Preferences and Associations with Demographic Characteristics

**DOI:** 10.3390/ijerph15020179

**Published:** 2018-01-23

**Authors:** Elizabeth A. Fradgley, Christine L. Paul, Jamie Bryant, Alison Zucca, Christopher Oldmeadow

**Affiliations:** 1 Priority Research Centre for Cancer Research, Innovation and Translation, University of Newcastle, Callaghan, NSW 2308, Australia; chris.paul@newcastle.edu.au; 2 Priority Research Centre for Health Behaviour, Hunter Medical Research Institute, University of Newcastle, Callaghan, NSW 2308, Australia; Jamie.bryant@newcastle.edu.au (J.B.); alison.zucca@newcastle.edu.au (A.Z.); 3 Health Behaviour Research Group, University of Newcastle, Callaghan, NSW 2308, Australia; 4 Hunter Medical Research Institute, HMRI Building, University of Newcastle, Callaghan, NSW 2308, Australia; christopher.oldmeadow@newcastle.edu.au; 5 School of Mathematical and Physical Sciences, Faculty of Science and Information Technology, University of Newcastle, Callaghan, NSW 2308, Australia

**Keywords:** health services, outpatient, chronic disease, cancer or neoplasm, quality improvement, consumer participation

## Abstract

Efficient patient-centred quality improvement requires an understanding of the system-wide areas of dissatisfaction along with evidence to identify the programs which can be strategically targeted according to specific patient characteristics and preferences. This cross-sectional study reports the proportion of chronic disease outpatients selecting 23 patient-centred improvement initiatives. Using univariate tests and multivariable logistic regressions, this multi-site study also identifies initiatives differentially selected by outpatients according to clinical and demographic characteristics. A total of 475 outpatients participated (49% response). Commonly selected initiatives included: reducing wait-times (22.3%); convenient appointment scheduling (16.0%); and receiving up-to-date treatment information (16.0%). Within univariate tests, preferences for information and service accessibility initiatives were not significantly associated with specific subgroups. However, seven initiatives were preferred according to age, gender, diagnosis status, and chronic disease type within multivariate models. For example, neurology outpatients were more likely to select assistance to manage psychological symptoms when compared to oncology outpatients (OR: 2.89). Study findings suggest that system-wide programs to enhance information provision are strategic approaches to improve experiences across patient characteristics. Furthermore, a few initiatives can be targeted to specific groups and emphasized the importance of detailed scoping analyses and tailored implementation plans when designing patient-centred quality improvement programs.

## 1. Introduction

Patient-centred care (PCC) is defined as care that is respectful of and responsive to individual patient values and needs [1]. In 2001, the Institute of Medicine (IOM) proposed PCC as one of six essential components of high quality healthcare [2]. Following the principles of PCC, patients are considered expert information sources and healthcare quality must in part be defined and evaluated according to patient perspectives and expectations for health services [3]. Consumer involvement in evaluating health services is widely recognized as important for promoting PCC and is increasingly included in international and Australian guidelines [4,5,6]. There are several ways in which consumers can be engaged in evaluating the quality of care, ranging from individual advocacy roles to groups of health service users responding to cross-sectional surveys about their experiences [7]. This latter form of involvement can be an inclusive and quick approach to gaining multiple perspectives on the quality of care. 

Data from large-scale patient surveys indicate that healthcare experiences are mediated by individual demographic and clinical characteristics [8,9,10]. While the relationship is admittedly complex, associations between experiences and patient characteristics have been reported in surveys of people with chronic diseases including cancer, heart disease, diabetes, and hypertension [9,10]. The difference in experiences suggests that efficient implementation of PCC for all patients requires not only an understanding of the common and system-wide areas of dissatisfaction, but also potential person- or group-specific concerns. In practice, targeting initiatives according to patient characteristics and preferences may be an efficient use of quality improvement resources. Quality improvement can pose financial or opportunity costs, whereby health services may be required to invest in infrastructure changes or reallocate clinical time to additional staff training or evaluation. While a targeted approach may be an efficient approach to improve the experiences of a specific group of patients, those initiatives valued by large proportions of service users independent of clinical or demographic characteristics may be appropriate to implement on a generic, system-wide level. 

While patient-experience surveys are important to highlight the gaps in the quality of care received, there are a number of limitations in using these data to inform health service improvement. Firstly, many of these tools do not directly request patients to identify their preferences for specific quality improvement initiatives. Without a clear and actionable improvement message, patient-experience surveys may be unable to stimulate health service change [11,12,13,14]. Secondly, large satisfaction or unmet need surveys are often conducted with very heterogeneous samples and therefore may obscure the details of patient preferences or experiences of particular subgroups. Conversely, surveys which include small sample sizes or a single patient group do not facilitate between-group statistical comparisons. Therefore, there is a need for studies which involve a sufficient number of patients across two or more chronic diseases with some commonality of experience (e.g., similar geographic location and health district) in order to advance our understanding of how patient characteristics may guide attempts to improve PCC in either a system-wide or targeted approach. 

The touch-screen Consumer Preference Survey (Consumer-PS) was designed to enable patients to directly inform quality improvement activities by selecting specific initiatives relevant to their experiences in chronic disease outpatient settings. The survey also allows for comparisons across a range of patient characteristics and chronic diseases as it can be quickly administered to a large and diverse sample with broadly-scoped PCC quality initiatives [15]. This is a novel approach to consumer engagement in quality improvement and expands upon existing patient-experience tools which do not allow respondents to directly select initiatives. This cross-sectional study reports the demographic and clinical factors associated with selecting particular quality improvement initiatives using the Consumer-PS.

This multisite study aims to report the:

1. Proportion of individuals selecting each quality improvement initiative; and,
2. Initiatives that are differentially selected between individuals according to demographic and clinical characteristics.

This is some of the first work to provide a broadly-scoped view of initiatives across chronic disease groups in tertiary outpatient care. For health services and policy developers, this information provides a set of generic initiatives that are equally valued across a range of health service users, along with a set of patient characteristics towards which quality improvement may be efficiently targeted. Briefly, this study concluded that information-based and accessibility initiatives were commonly selected by outpatients regardless of demographic characteristics; however, a few targetable initiatives emerged, particularly for newly-diagnosed outpatients and neurology outpatients.

## 2. Methods

Study design: A cross-sectional survey of outpatients was conducted according to the Strengthening the Reporting of Observational Studies in Epidemiology (STROBE) statement [16]. Ethics approval was provided by Hunter New England Human Research Ethics Committee (HREC 12/08/15/4.04) and the University of Newcastle Human Research Ethics Committee (H-2013-0234). Data was collected over a 16-month period. 

Setting: Outpatients were recruited from two publically-funded tertiary hospitals located in one health district within New South Wales, Australia. This included a specialist clinic providing cardiology or neurology care and a medical oncology centre providing physician consultation and intravenous chemotherapy treatment. 

Participants: English-speaking adult outpatients were recruited from clinic waiting rooms or treatment areas by trained research assistants. To be eligible, participants had to have attended the clinic at least once prior to recruitment and were asked to reflect upon this experience whilst completing surveys. Assistance with touch-screen devices was provided as needed. Research assistants estimated the gender and age-range of non-consenters to ascertain possible consent bias. 

Measurement: Participants completed the Consumer-PS and a patient characteristic module. 

**Figure 1:**
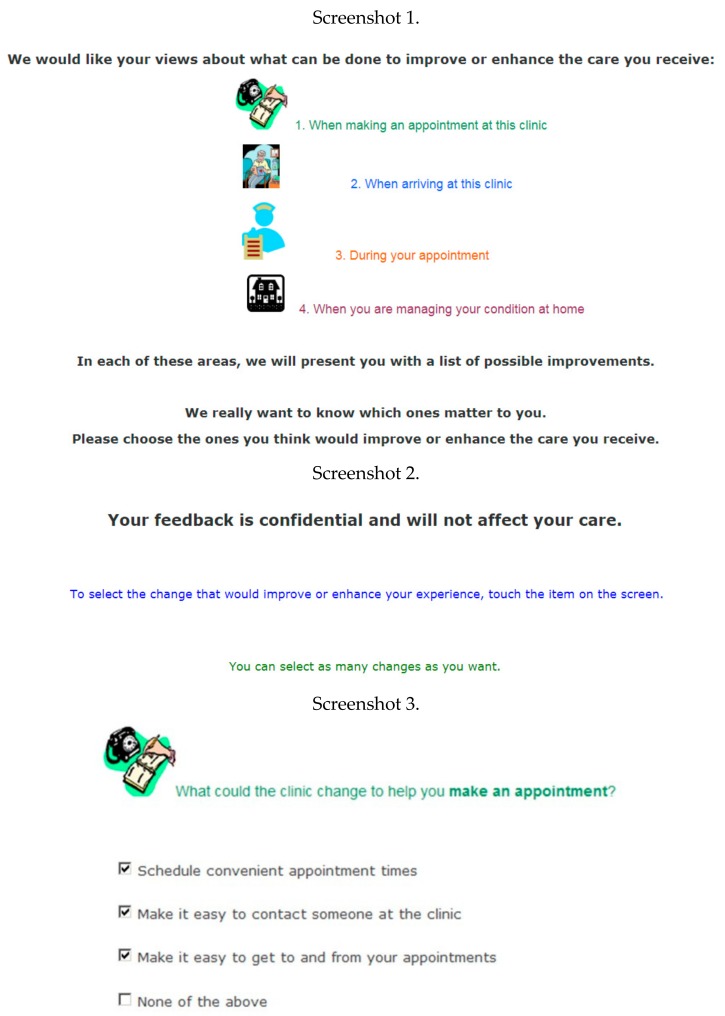
Screenshots of the Consumer-PS instructions and survey items.

The Consumer-PS: The Consumer-PS includes 23 PCC quality improvement initiatives which were developed based on a structured literature review, and iterative consultation with two groups of service providers (*n* = 20) and consumers (*n* = 27) [15]. During the initial development and refinement process, each initiative was mapped to one of eight dimensions of PCC: access to care and services; coordination and integration; emotional support; information, education, and communication; involvement of family and close others; transition and continuity of care; physical comfort; and respect for patients’ preferences, values, and needs. However, following consumer feedback, the survey content was grouped into four topics related to how an outpatient may sequentially experience care: (1) making or coordinating an appointment; (2) arriving and accessing the clinic; (3) during an appointment and consultation; and (4) self-management at home [15]. Respondents were instructed by research assistants to reflect on their previous interaction(s) within the clinic and select the changes that would improve their experience. Respondents could select as many changes as desired or none. A screenshot of the Consumer-PS introduction screen and example of the survey items are provided in Figure 1.

A validation study reported that the average observed agreement across the 23 initiatives was 93.7% with moderate or substantial test-retest reliability (Cohen’s kappa = 0.44–0.1.0, observed percentage agreement = 79.5–100.0%); the study also reported the high levels of respondent acceptability [15]. For example, 98% of respondents indicated that the survey was easy to complete and 85% felt the survey helped them to decide which initiatives were of most importance to them. On average, the Consumer-PS takes approximately nine minutes to complete and requires a Flesch-Kincaid reading level of grade 7 [15]. The Consumer-PS also includes 110 specific initiatives available via adaptive questioning and a relative prioritisation exercise. The data from these two modules is presented elsewhere [17]. 

The patient characteristic module included the following:

- Demographic information: date of birth; gender; marital status (single or married/de-facto partner); highest education level achieved (high school equivalent of year 10 or lower, high school completion, diploma or trade certificate, Bachelor’s or postgraduate degree); Aboriginal and or Torres Strait Islander origin; private insurance coverage; and possession of a concession card. Australian concession cards reduce healthcare costs and are restricted to pensioners, social security allowance recipients, and low-income earners.
- Clinical characteristics: reason for attending the clinic (a routine exam for a diagnosed condition; discussion of symptoms for a diagnosed or non-diagnosed condition; or to receive tests or treatments); and appointment frequency within the last six months. Oncology participants completed two additional questions, relating to primary cancer site and time since receiving diagnosis, if known. Previous research with cancer patients has reported varying information and supportive care needs based on time since diagnosis and tumour type, and therefore may impact the preference for these types of initiatives [18].

Statistical methods: Descriptive statistics reported demographic and clinical variables and the proportion of individuals selecting each initiative. To identify initiatives that are differentially selected according to these variables, a two-step process was completed:

- Univariate Chi Square or Fisher’s Exact tests were used to compare the proportion selecting an initiative between demographic or clinical subgroups. To reduce the number of spuriously reported associations due to the large number of initiatives tested (*n* = 23), a stringent Bonferroni significance threshold of 0.002 was used. Initiatives that reached this threshold for at least one demographic or clinical variable proceeded to the next stage;
- Multivariable logistic regression was used to identify the characteristics associated with selecting initiatives. All demographic variables were included in the multivariable model, and variables were removed from the models if the Wald *p*-values were greater than 0.25 and removal from the model did not alter the remaining coefficients by more than 15% [19]. Adjusted differences between subgroups in the probability of selecting an item are presented on the odds ratio scale.

All data analysis was completed using Stata 11 (Statacorp, College Station, TX, USA).

## 3. Results

Participants: A total of 968 individuals were approached to participate, of which 608 individuals consented to complete touch screen surveys (62.8% consent). A total of 475 (78.1%) participants completed the survey, of which 271 (57.1%) were oncology outpatients, 135 (28.7%) were neurology outpatients, and 68 (14.4%) were cardiology outpatients. 

Sample demographic and clinical characteristics are available in Table 1. On average, participants were 60 years of age, married or living with partner (66.7%), did not have private health insurance (59.2%), and had completed the high school equivalent of a year ten or lower level of education (52.6%). The sample had an equal ratio of men (50.2%) to women (49.8%).

Differences in the characteristics of those who consented versus those who declined to participate, as well as those who completed a survey versus those who did not, are available in the Supplementary Materials (Tables S1 and S2). Briefly, individuals older than 71 years reported lower consent and completion rates (59.9% and 48.1%, respectively). Men, medical oncology outpatients, and those attending for tests or treatment reported higher completion rates. 

The proportion of individuals selecting each PCC initiative: Across the 23 initiatives, improved car parking was selected by the greatest proportion of respondents (67.2%), followed by reduced wait-times (22.3%), up-to-date information provision (16.2%), and convenient appointment scheduling (16.0%) (Table 2). Comfortable waiting rooms and comfortable treatment rooms were selected by a small proportion of participants (1.7% and 1.5%, respectively). 

Initiatives selected by statistically similar proportions across all patient characteristics: Sixteen initiatives were selected by statistically similar proportions (*p*-values > 0.002) across all demographic and clinical characteristics (Table 2).

Initiatives selected by statistically different proportions across at least one patient characteristic: Seven initiatives were selected by varying proportions of participants (*p*-values ≤ 0.002), according to demographic or clinical characteristics (Table 2). 

**Adjusted odds of selecting an initiative according to patient characteristics:** Table 3, Table 4, Table 5, Table 6, Table 7, Table 8 and Table 9 provide results from the multivariable logistic regression models used to compare demographic and clinical factors for the seven initiatives, reporting significant differential proportions. The results of the final models are summarised below; variables were removed from the models if the Wald *p*-values were greater than 0.25 and removal from the model did not alter the remaining coefficients by more than 15% [19].

*Provide more information on treatment and condition during clinical appointment:* Individuals who were attending the clinic to discuss an undiagnosed condition were more than four times (OR: 4.17, 95% CI: 1.21–14.4) likely to select receiving additional information during an appointment than those attending to discuss a diagnosed condition (Table 3).

*Ensure concerns are discussed with health professionals:* Females (OR: 3.05, 95% CI: 1.46–6.37) and those with a high school education (OR: 2.72, 95% CI: 1.01–7.35) were approximately three times more likely to select this initiative (Table 4).

*Assistance/information to manage emotional symptoms*: Neurology outpatients were more likely (OR: 2.89, 95% CI: 1.37–6.10) to select assistance and information to manage emotional symptoms compared to medical oncology outpatients (Table 5). Those without private insurance (OR: 0.49, 95% CI: 0.23–1.01) and, compared to individuals between the ages of 18 and 46.9 years, those between the ages of 60–66.9 years (OR: 0.09, 95% CI: 0.01–0.78), were less likely to select this initiative.

*Information and assistance with finances, work leave, and insurance*: The odds of selecting this initiative were significantly associated with age (Table 6). Compared to individuals between 18 and 47 years of age, those between the ages of 60 and 66 years were less likely to select this initiative (OR: 0.13, 95% CI: 0.30–0.63). No individuals 67 years of age and older or those who attended a clinic to discuss symptoms or tests for an undiagnosed condition selected this initiative.

*Make it easier to contact the clinic:* Women reported significantly greater odds (OR: 2.53, 95% CI: 1.44–4.46) of selecting this initiative compared to men (Table 7).

*Improved hospital catering:* The odds of selecting improved catering were greater for individuals: with post-secondary educations (OR: 2.57; 95% CI: 0.99–6.67); attending for tests or treatments (OR: 4.83, 95% CI: 1.29–18.04); and who attended clinics more frequently (OR: 1.41, 95% CI: 1.00–1.99) in the past six months (Table 8). Compared to individuals between 18 and 47 years of age, those between the ages of 67 and 74.9 years were less likely to select this initiative (OR: 0.30, 95% CI: 0.09–1.00).

*Improved hospital parking:* Neurology and cardiology outpatients were less likely (OR: 0.25, 95% CI: 0.14–0.45; OR: 0.32, 95% CI: 0.17–0.64, respectively) to select improved parking as compared to oncology outpatients (Table 9). Uninsured individuals (OR: 0.48, 95% CI: 0.30–0.77) and those between the ages of 60 and 66.9 years were less likely to select improved parking (OR: 0.47, 95% CI: 0.24–0.95).

## 4. Discussion

**Figure 2:**
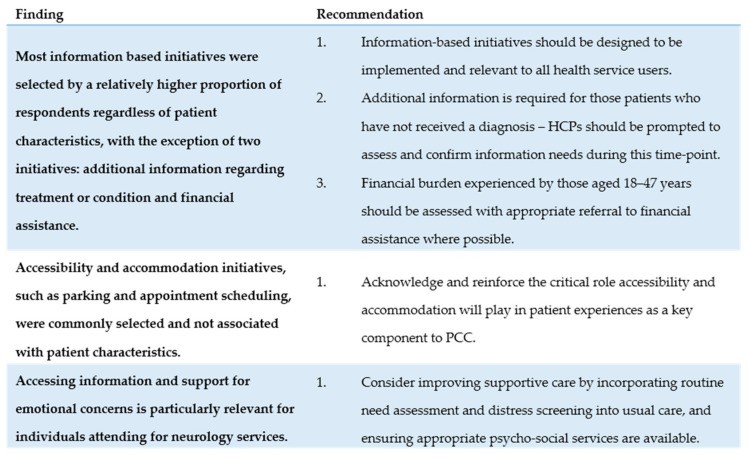
Summary of findings and related recommendations for health services and professionals.

This study suggests that several commonalities exist in the quality improvement initiatives identified by participants across demographic and clinical variables, which may warrant system-wide implementation. However, a few initiatives may be only appropriate in particular clinics and should be strategically targeted to specific patient groups. With the shift towards providing multiple types of specialized care in a single, centralized, and high-volume facility, it is increasingly important to consider how a diverse group of patients may experience care within a single setting [20,21]. Clear targets for improvement might only emerge when exploring how specific patient groups may experience care. Service recommendations based on these findings are presented below, and are summarised within Figure 2. 

Information-based initiatives may warrant system-wide implementation: Commonly selected initiatives included being kept up-to-date on treatment and condition progress (16.2%), access to information at home (14.1%), information to manage emergencies (11.4%), and assistance and information to maintain activities of daily living (10.3%). Given the relatively higher frequency at which these initiatives were selected by study participants, health services should design programs and policies to improve information provision that can be implemented on a system-wide level. 

The proportion of individuals selecting information-based initiatives did not differ significantly across demographic or clinical characteristics, with the exception of two characteristics. Firstly, the odds of selecting assistance or information on financial assistance was significantly associated with age; compared to individuals between 18 and 47 years of age, those between the ages of 60 and 66 years were less likely to select this initiative. This finding highlights the need to consider the financial burden experienced by individuals who suffer income-loss as a result of illness. 

Secondly, relative to individuals with a diagnosed condition, individuals who were attending an appointment for an undiagnosed condition were approximately four times more likely to select additional information on treatment or condition. This finding of high information needs prior to or at the time of diagnosis aligns with closely existing studies, including systematic reviews involving cancer patients [22], qualitative research with individuals experiencing undiagnosed chest pain [23], and cross-sectional surveys of individuals with epilepsy [24]. As previous research suggests healthcare professionals may underestimate the amount of information desired by patients [25], embedded prompts to assess and respond to information needs at time of diagnosis may be beneficial and assist health services to address this patient-perceived area of need. 

Initiatives to improve the accessibility and accommodation of care were commonly selected: While patient concerns regarding the accommodation and amenities of care are not clinical issues, health services need to consider how such infrastructure and organizational factors may influence perceptions of care. A literature review of psycho-oncology need assessment tools suggests that health professionals may consider some patient concerns relating to these more front-line areas as outside their scope of practice [26]; furthermore, health service infrastructure may not be easily modified by healthcare professionals. However, hospital physical environments and organisation have been long-associated with patients’ wellbeing, with previous research suggesting health services can be a ‘healing landscape’ or cause additional distress for patients and families [27,28]. The role of the hospital environment is also acknowledged in the Planetree Model of Patient-Centered Care [29,30].

Within this study, reduced wait-times (22.3%) and convenient appointment scheduling systems (16.0%) were commonly selected by participants and were not associated with patient characteristics. O’Brien et al. [31] suggested that health services should inform patients of expected delays and reasons for this delay when receiving oncology outpatient treatment. When informed of the reasons behind lengthy wait-times, patients are more accurate in gauging the actual time spent in clinic wait rooms [32]. This may be a relatively simple solution as opposed to altering staff-to-patient ratios and other organisational changes in the form of appointment scheduling models. 

The accessibility of care remains an essential component to PCC and practical barriers to navigating health services can have long term patient and system implications in the form of patients delaying or limiting service use or creating additional distress. While the Consumer-PS data relating to organisational and infrastructure improvement may not directly influence healthcare professionals’ personal practice, these findings can provide the needed data to lobby for infrastructure changes with health service administrators and directors. It is also important to note that service evaluation rarely includes collecting and reviewing patient-reported data on perceived quality and accessibility of the care environment [33]. Without robust data on the value of these non-clinical aspects of care, it may be difficult to rationalize the cost and time spent on improving these areas. However, accommodating patient preferences is a fundamental tenet of PCC and therefore a necessary component of high quality healthcare. 

Quality improvement is not a one-size fits all approach and preferences are influenced by demographic factors: This study sought to identify the initiatives selected by different proportions of individuals and the adjusted odds of selecting these initiatives according to demographic and clinical characteristics. For the seven initiatives significantly associated with patient characteristics, several key characteristics emerged in multivariate models including: age, gender, chronic condition, education, reason for attending, appointment frequency, and health insurance coverage. 

Increasing age and male gender was frequently associated with lower odds of selecting quality improvement initiatives. Within this study, compared to the youngest age group, older age groups reported decreased odds of selecting: access to information and assistance for financial, work, or insurance concerns; improved catering; information to manage emotional symptoms; and improved parking. Additionally, women were three times more likely to select being able to discuss concerns with a health professional and 2.5 times more likely to select ease of contacting the clinic. 

Previous studies have reported differences in patient satisfaction according to both gender and age. Results from the 2011/2012 English Cancer Patient Experience Survey, a national mail-out survey completed by 71,793 individuals, found that individuals aged 65–74 years reported a more positive experience [8]. Concordant with our results, women were also significantly more likely to report a comparatively poorer experience and similarly reported worse experiences when attempting to contact a clinical nurse specialist or being able to discuss worries and fears with staff. However, inconsistent with our results, this study found that those in the eldest group (85 years or more) reported worse experiences. Less than 8% of our sample was within this advanced age range and it is possible that the views of this smaller subgroup are masked within the broader age group of 75 years and plus. While it is beyond the quantitative results of this study, previous research indicates that elderly individuals are less likely to indicate poor perceptions of care due to greater experience with navigating healthcare services (i.e., maturation explanation) and lower expectations based on generational values [34]. 

Accessing information and support for emotional concerns is particularly relevant for individuals attending for neurology services. As compared to medical oncology outpatients, neurology outpatients were significantly more likely to select emotional support (OR = 2.89, *p* = 0.005). High levels of unmet emotional needs have been previously reported by individuals with neurological conditions, such as stroke and multiple sclerosis. For example, a study of long-term needs in individuals up to five years post-stroke reported that approximately 77% reported emotional problems, with the majority indicating that these needs went largely unmet [35]. A recent systematic review of individuals with multiple sclerosis highlighted the importance of individuals’ emotional experiences of care, particularly at time of diagnosis, with poor information provision and limited access to supportive care services associated with increased patient distress [36]. Health services specializing in neurological care may consider improving supportive care by incorporating routine need assessment and distress screening into usual care, and ensuring that appropriate psycho-social services are available. These approaches have been successfully associated with improved supportive oncology care [37]. 

Limitations: It is possible that the study results are influenced by a social desirability bias in that participants may have been unwilling to indicate discontent with healthcare service and the results may thus demonstrate a ceiling effect. However, these results follow a similar trend identified within satisfaction surveys [38]. 

This study followed a two-step analysis to identify a concise list of patient characteristics towards which specific quality improvement initiatives may be efficiently targeted. Due to the large number of tests, a stringent Bonferroni threshold was used to determine statistical significance at the univariate level. While this reduced the potential of results being influenced by a Type II error, an association between a patient factor and quality improvement preference may have been missed. 

This study included a limited set of variables to describe patient characteristics and clinical settings. Individuals were asked to report if they possessed an Australian concession card and this provides a very rough estimate of individuals’ socioeconomic status. While this variable could have been better recorded, the association between patient experiences and social disadvantage is well-documented [39]. Capturing additional variables, such as staff volumes and available service amenities, would have been valuable to explore the association between clinic settings and preferred initiatives. A more complete description of participating clinics using these variables would also have been informative for evaluating the degree to which these results are generalizable to other services. Finally, due to consent and completion biases, replication in additional sites would strengthen the validity and representativeness of the results. 

The next steps: This study presented data from health services interested in pursuing those quality improvement initiatives preferred by a cross-section of their patients. The next step within the quality improvement cycle is to use these results to design and implement an improvement program; this work is currently underway in a stepped wedge cluster randomised controlled trial evaluating a consumer driven breakthrough action model in improving aspects of cancer treatment systems (Trial registration: ACTRN12614000702617). The results from the larger study will provide additional information on how the Consumer-PS, or similar surveys, can be used as a means to incorporate consumer perspectives into evaluating and improvement the delivery of PCC. 

## 5. Conclusions

In order to improve the quality of outpatient chronic disease care according to patients’ preferences and priorities, health services should focus on implementing information-based initiatives on a system-wide level. However, a few targetable initiatives emerged, such as additional emotional support for neurology outpatients. Given the number of factors associated with patient preferences’ for quality improvement, this study emphasizes the need for detailed scoping analyses to inform any quality improvement and specific concerns need to be addressed using a more tailored approach. 

## Figures and Tables

**Table 1:** Sample demographic and clinical characteristics (*n* = 475).

Sample Characteristics			Number of Participants (%)
Average years of age			average = 60.3 (SD 15.6)
Male			238 (50.1)
Highest level of education attained			
	High school equivalent of year 10 or lower		250 (52.6)
	High school completion		53 (11.2)
	Diploma or trade certificate		104 (21.9)
	Bachelor’s or postgraduate degree		68 (14.3)
Marital status			
	Married or living with partner		317 (66.7)
	Single (never married, divorced or widowed)		158 (33.3)
Aboriginal and/or Torres Strait Islander origin			19 (4.0)
No private health insurance coverage			281 (59.2)
Concessional card			323 (68.0)
Recruited from			
	Cardiology or neurology		204 (42.9)
		Cardiology-specific	68 (33.5)
		Neurology-specific	135 (66.5)
	Medical oncology		271 (57.1)
Reason for attending			
	To discuss symptoms, treatments or tests for diagnosed condition		90 (19.0)
	To discuss symptoms or tests for undiagnosed condition		36 (7.6)
	To receive tests or treatments for diagnosed condition		125 (26.3)
	For a routine exam for a diagnosed condition		215 (45.3)
	Do not know		9 (1.9)
Number of appointments in last three months			
	At least once in the last six months		253 (53.3)
	2–3		95 (20.0)
	4–5		62 (13.1)
	6+		65 (13.7)
Primary cancer site (*n* = 271)			
	Breast		60 (22.1)
	Bowel		33 (12.2)
	Blood		53 (19.6)
	Prostate		19 (7.0)
	Gynaecological		12 (4.4)
	Head and neck		12 (4.4)
	Lung		22 (8.1)
	Melanoma		2 (0.7)
	Other		39 (14.4)
	Do not know		7 (2.6)
Time since cancer diagnosis (*n* = 271)			
	Less than 6 months		66 (24.6)
	Between 6–12 months		52 (19.2)
	Between 1–3 years		59 (21.8)
	More than 3 years		81 (29.9)
	Do not know		13 (4.8)

**Table 2:** Proportion of participants selecting each initiative and proportional differences according to patient characteristics identified

Initiatives	Number of Participants (%)	According to Patient Characteristics, Selected by:	
		Similar Proportions ^1^	Different Proportions
Reduce waiting times	106 (22.3)	✔	
Keep you up-to-date on treatment and condition progress	77 (16.2)	✔	
Provide more convenient appointment scheduling and times	76 (16.0)	✔	
Access to information to review at home	67 (14.1)	✔	
Information on how to manage medical emergencies	54 (11.4)	✔	
Assistance/information to maintain activities of daily living	49 (10.3)	✔	
Assistance/information to manage physical symptoms	45 (9.5)	✔	
Involve you in treatment decisions	32 (6.7)	✔	
Help to arrange transport to and from the clinic	32 (6.7)	✔	
Ensure good interactions with all clinic staff	31 (6.5)	✔	
Better coordination of your care	27 (5.7)	✔	
Access to help or information for family support	26 (5.5)	✔	
Minimize pain or discomfort during treatment	17 (3.6)	✔	
Ensure family/friends are comfortable in wait-rooms	9 (1.9)	✔	
Provide a comfortable and pleasant waiting room	8 (1.7)	✔	
Provide a comfortable and pleasant treatment room	7 (1.5)	✔	
Improve car parking	319 (67.2)		✔
Make it easier to contact the clinic	67 (14.1)		✔
Assistance/information to manage emotional symptoms	44 (9.3)		✔
Ensure concerns are discussed with health professionals	42 (8.8)		✔
Improve hospital catering	41 (8.6)		✔
Provide more treatment/condition information during visit	25 (5.3)		✔
Assistance/information relating to finance, work, insurance	24 (5.1)		✔

^1^ Chi-Square or Fisher’s Exact tests are not significant (*p*-values > 0.002).

**Table 3:** Adjusted odds of selecting “Provide more information on treatment and condition during a clinical appointment”.

Characteristics		Adjusted OR (95% CI)	*p*-Value
Highest level of education attained			
	High school equivalent of year 10 or lower	Reference	
	High school completion	1.99 (0.58–6.81)	0.27
	Diploma or trade certificate	1.23 (0.43–3.58)	0.69
	Bachelor’s or postgraduate degree	0.77 (0.16–3.66)	0.74
Reason for attending ^1^			
	To discuss symptoms, treatments or tests for diagnosed condition	Reference	
	To discuss symptoms or tests for undiagnosed condition	4.17 (1.21–14.40)	0.02
	To receive tests or treatments for diagnosed condition	0.46 (0.11–1.92)	0.29
	For a routine exam for a diagnosed condition	0.56 (0.17–1.84)	0.34
Appointment frequency in the last 6 months (continuous)		1.21 (0.78–1.86)	0.40

^1^ 5 individuals (0.9%) did not know the reason for attendance and were excluded from analysis.

**Table 4:** Adjusted odds of selecting “Ensure concerns are discussed with health professionals”.

Characteristics		Adjusted OR (95% CI)	*p*-Value
Gender			
	Male	Reference	
	Female	3.05 (1.46–6.37)	0.003
Highest level of education attained			
	High school equivalent of year 10 or lower	Reference	
	High school completion	2.72 (1.01–7.35)	0.05
	Diploma or trade certificate	2.04 (0.87–4.77)	0.10
	Bachelor’s or postgraduate degree	2.00 (0.7–5.30)	0.17
Recruited from			
	Medical oncology clinic	Reference	
	Neurology clinic	0.53 (0.22–1.30)	0.16
	Cardiology clinic	0.68 (0.23–2.06)	0.50
Reason for attending ^1^			
	To discuss symptoms, treatments or tests for diagnosed condition	Reference	
	To discuss symptoms or tests for undiagnosed condition	3.05 (0.91–10.27)	0.07
	To receive tests or treatments for diagnosed condition	0.74 (0.23–2.39)	0.61
	For a routine exam for a diagnosed condition	0.89 (0.33–2.42)	0.82

^1^ 5 individuals (0.9%) did not know the reason for attendance and were excluded from analysis.

**Table 5:** Adjusted odds of selecting “Assistance or information to manage emotional symptoms”.

Characteristics		Adjusted OR (95% CI)	*p*-Value
Age percentile (years)			
	1–20 (18–46.9)	Reference	
	21–40 (47–59.9)	1.72 (0.72–4.13)	0.22
	41–60 (60–66.9)	0.51 (0.16–1.57)	0.24
	61–80 (67–74.9)	0.09 (0.01–0.78)	0.03
	80–100 (75+)	0.54 (0.18–1.63)	0.28
Highest level of education attained			
	High school equivalent of year 10 or lower	Reference	
	High school completion	2.34 (0.88–6.3)	0.09
	Diploma or trade certificate	1.67 (0.72–3.91)	0.24
	Bachelor’s or postgraduate degree	1.13 (0.33–3.29)	0.81
Recruited from			
	Medical oncology clinic	Reference	
	Neurology clinic	2.89 (1.37–6.10)	0.005
	Cardiology clinic	0.98 (0.27–3.62)	0.98
Health insurance coverage			
	Private health insurance	Reference	
	No private health insurance coverage	0.49 (0.23–1.01)	0.05
Possesses an Australian concession card			
	Yes	Reference	
	No	0.99 (0.47–2.09)	0.97

**Table 6:** Adjusted odds of selecting “Access to information and assistance for insurance, work leave and finances”.

Characteristics		Adjusted OR (95% CI)	*p*-Value
Age percentile (years)			
	1–20 (18–46.9)	Reference	
	21–40 (47–59.9)	0.62 (0.24–1.60)	0.32
	41–60 (60–66.9)	0.13 (0.03–0.63)	0.01
	61–80 (67–74.9)	Omitted	
	80–100 (75+)	Omitted	
Highest level of education attained			
	High school equivalent of year 10 or lower	Reference	
	High school completion	1.33 (0.32–5.55)	0.69
	Diploma or trade certificate	2.02 (0.70–5.80)	0.19
	Bachelor’s or postgraduate degree	1.29 (0.36–4.57)	0.71
Reason for attending ^1^			
	To discuss symptoms, treatments or tests for diagnosed condition	Reference	
	To discuss symptoms or tests for undiagnosed condition	Omitted	
	To receive tests or treatments for diagnosed condition	1.64 (0.43–6.32)	0.47
	For a routine exam for a diagnosed condition	1.13 (0.34–3.81)	0.84

^1^ 5 individuals (0.9%) did not know the reason for attendance and were excluded from analysis.

**Table 7:** Adjusted odds of selecting “Make it easier to contact the clinic”.

Characteristics		Adjusted OR (95% CI)	*p*-Value
Gender			
	Male	Reference	
	Female	2.53 (1.44–4.46)	0.001
Highest level of education attained			
	High school equivalent of year 10 or lower	Reference	
	High school completion	2.06 (0.93–4.53)	0.07
	Diploma or trade certificate	1.10 (0.53–2.28)	0.80
	Bachelor’s or postgraduate degree	2.01 (0.95–4.27)	0.07
Recruited from			
	Medical oncology clinic	Reference	
	Neurology clinic	1.84 (0.96–3.55)	0.07
	Cardiology clinic	0.79 (0.31–2.04)	0.63
Reason for attending ^1^			
	To discuss symptoms, treatments or tests for diagnosed condition	Reference	
	To discuss symptoms or tests for undiagnosed condition	0.91 (0.32–2.53)	0.85
	To receive tests or treatments for diagnosed condition	0.41 (0.16–1.09)	0.07
	For a routine exam for a diagnosed condition	0.69 (0.35–1.36)	0.28

^1^ 5 individuals (0.9%) did not know the reason for attendance and were excluded from analysis.

**Table 8:** Adjusted odds of selecting “Improved hospital catering”.

Characteristics		Adjusted OR (95% CI)	*p*-Value
Age percentile (years)			
	1–20 (18–46.9)	Reference	
	21–40 (47–59.9)	0.51 (0.18–1.39)	0.19
	41–60 (60–66.9)	0.58 (0.20–1.64)	0.30
	61–80 (67–74.9)	0.30 (0.09–1.00)	0.05
	80–100 (75+)	0.33 (0.10–1.10)	0.73
Highest level of education attained			
	High school equivalent of year 10 or lower	Reference	
	High school completion	1.15 (0.35–3.80)	0.82
	Diploma or trade certificate	1.77 (0.74–4.25)	0.20
	Bachelor’s or postgraduate degree	2.57 (0.99–6.67)	0.05
Reason for attending ^1^			
	To discuss symptoms, treatments or tests for diagnosed condition	Reference	
	To discuss symptoms or tests for undiagnosed condition	0.62 (0.06–6.47)	0.69
	To receive tests or treatments for diagnosed condition	4.83 (1.29–18.04)	0.02
	For a routine exam for a diagnosed condition	1.92 (0.52–7.05)	0.33
Appointment frequency in the last 6 months (continuous)		1.41 (1.00–1.99)	0.05
Health insurance coverage			
	Private health insurance	Reference	
	No private health insurance coverage	2.10 (0.96–4.60)	0.06

^1^ 5 individuals (0.9%) did not know the reason for attendance and were excluded from analysis.

**Table 9:** Adjusted odds for selecting “Improved hospital parking”.

Characteristics		Adjusted OR (95% CI)	*p*-Value
Age percentile (years)			
	1–20 (18–46.9)	Reference	
	21–40 (47–59.9)	0.70 (0.36–1.37)	0.30
	41–60 (60–66.9)	0.48 (0.24–0.95)	0.04
	61–80 (67–74.9)	0.74 (0.37–1.50)	0.41
	80–100 (75+)	0.56 (0.28–1.12)	0.10
Highest level of education attained			
	High school equivalent of year 10 or lower	Reference	
	High school completion	0.83 (0.43–1.61)	0.59
	Diploma or trade certificate	1.36 (0.78–2.37)	0.28
	Bachelor’s or postgraduate degree	0.60 (0.32–1.13)	0.11
Recruited from			
	Medical oncology clinic	Reference	
	Neurology clinic	0.25 (0.14–0.45)	<0.001
	Cardiology clinic	0.32 (0.17–0.64)	0.001
Reason for attending ^1^			
	To discuss symptoms, treatments or tests for diagnosed condition	Reference	
	To discuss symptoms or tests for undiagnosed condition	0.81 (0.34–1.92)	0.34
	To receive tests or treatments for diagnosed condition	0.64 (0.30–1.36)	0.30
	For a routine exam for a diagnosed condition	1.21 (0.69–2.10)	0.69
Appointment frequency in the last 6 months (continuous)		1.17 (0.93–1.48)	0.18
Health insurance coverage			
	Private health insurance	Reference	
	No private health insurance coverage	0.48 (0.30–0.77)	0.002

^1^ 5 individuals (0.9%) did not know the reason for attendance and were excluded from analysis.

## Supplementary Materials

The following are available online at http://www.mdpi.com/1660-4601/15/2/179/s1, Table S1: Demographic characteristics by consent status, with goodness of fit statistics, Table S2: Demographic characteristics by completion status, with goodness of fit statistics.
