# Supplementary material for: System-Wide and Group-Specific Health Service Improvements: Cross-Sectional Survey of Outpatient Improvement Preferences and Associations with Demographic Characteristics
Source: Int J Environ Res Public Health. 2018 Jan 23;15(2):179. doi: 10.3390/ijerph15020179 (PMC5858254; doi:10.3390/ijerph15020179)
Supplement: Supplementary file 1 [file ijerph-15-00179-s001.docx]

Supplementary Material: Consent and completion details

**Table S1.** Demographic characteristics by consent status, with goodness of fit statistics.

| **Characteristic** | | **Non-Consenters (%) (n = 283) ^1^** | **Consenters (%) (n = 608) ^1^** | ***Test Statistics*** | |
| --- | --- | --- | --- | --- | --- |
|  |  |  |  | **χ^2^ (*df*)** | ***P*** |
| Male (*n* = 410) | | 129 (45.6) | 281 (46.5) | 0.07 (1) | 0.79 |
| Clinic site | |  |  | 0.21 (2) | 0.65 |
|  | Medical oncology (*n* = 476) | 148 (52.3) | 328 (54.0) |  |  |
|  | Cardiology and neurology (*n* = 415) | 135 (47.7) | 280 (46.1) |  |  |
| Age category | |  |  | 12.8 (4) | .01 |
|  | 18–25 (*n* = 37) | 5 (1.8) | 32 (5.3) |  |  |
|  | 26–40 (*n* = 144) | 48 (17.0) | 96 (15.8) |  |  |
|  | 41–55 (*n* = 239) | 79 (27.9) | 160 (26.3) |  |  |
|  | 56–70 (*n* = 319) | 90 (31.8) | 229 (37.6) |  |  |
|  | ≥71 (*n* = 152) | 61 (21.5) | 91 (15.0) |  |  |

^1^. Data missing for 77 (7.9%) individuals.

**Table S2.** Demographic characteristics by completion status, with goodness of fit statistics.

| **Characteristic** | | **Incomplete (%) *n* = 195** | **Complete (%) *n* = 475** | ***Test Statistics*** | |
| --- | --- | --- | --- | --- | --- |
|  |  |  |  | **χ^2^ (*df*)** | ***P*** |
| Male (*n* = 296) | | 58 (37.2) | 238 (50.1) | 7.55 (1) | 0.006 |
| Clinic site | |  |  | 13.0(1) | <0.001 |
|  | Cardiology/neurology, public facility (*n* = 283) | 79 (60.7) | 204 (43.0) |  |  |
|  | Medical oncology, public facility (*n* = 322) | 51 (39.2) | 271 (57.1) |  |  |
| Age category | |  |  | 37.7 (4) | <0.001 |
|  | 18–25 (*n* = 33) | 11 (5.6) | 22 (4.6) |  |  |
|  | 26–40 (*n* = 108) | 37 (19.0) | 71 (15.0) |  |  |
|  | 41–55 (*n* = 163) | 34 (17.4) | 129 (27.2) |  |  |
|  | 56–70 (*n* = 233) | 49 (25.1) | 184 (38.8) |  |  |
|  | ≥71 (*n* = 133) | 64 (32.8) | 69 (14.5) |  |  |
| Highest education level | |  |  | 12.9(3) | 0.005 |
|  | High school equivalent of year 10 or lower (*n* = 316) | 66 (48.5) | 250 (53.6) |  |  |
|  | High school completion (*n* = 84) | 31 (22.8) | 53 (11.2) |  |  |
|  | Diploma or trade certificate (*n* = 130) | 26 (19.1) | 104 (21.9) |  |  |
|  | Bachelor’s or postgraduate degree (*n* = 81) | 13 (9.6) | 68 (14.3) |  |  |
| Reason for attending the clinic | |  |  | Fisher exact < 0.001 | |
|  | To discuss symptoms/treatments/tests, diagnosed (*n* = 117) | 27 (23.91) | 90 (19.2) |  |  |
|  | To discuss symptoms/tests, undiagnosed (*n* = 49) | 13 (11.5) | 36 (7.7) |  |  |
|  | To receive tests or treatments, diagnosed (*n* = 131) | 6 (5.3) | 125 (26.6) |  |  |
|  | For a routine exam, diagnosed (*n* = 278) | 63 (55.8) | 215 (45.7) |  |  |
